# Quality of Life in Brain Tumor Patients and Their Relatives Heavily Depends on Social Support Factors during the COVID-19 Pandemic

**DOI:** 10.3390/cancers13061276

**Published:** 2021-03-13

**Authors:** Fabian M. Troschel, Franziska Ahndorf, Lisa-Marie Wille, Ralf Brandt, Johanna Jost, Sylvia Rekowski, Hans Theodor Eich, Walter Stummer, Rainer Wiewrodt, Kathleen Jetschke, Dorothee Wiewrodt

**Affiliations:** 1 Department of Radiation Oncology, Münster University Hospital, 48149 Münster, Germany; hans.eich@ukmuenster.de; 2 Department of Neurosurgery, Münster University Hospital, 48149 Münster, Germany; Franziska.Ahndorf@ukmuenster.de (F.A.); Lisamarie.Wille@ukmuenster.de (L.-M.W.); Ralf.Brandt@ukmuenster.de (R.B.); johanna.jost@ukmuenster.de (J.J.); Walter.Stummer@ukmuenster.de (W.S.); 3 Department of Neurosurgery, University Hospital, Ruhr-Universität Bochum, 44892 Bochum, Germany; Sylvia.Rekowski@kk-bochum.de (S.R.); Kathleen.Jetschke@kk-bochum.de (K.J.); 4 Pulmonary Division, Department of Medicine A, Münster University Hospital, 48149 Münster, Germany; Rainer.Wiewrodt@ukmuenster.de

**Keywords:** COVID-19, brain tumors, glioblastoma, mental health, quality of life, caregivers, social support, depression, anxiety, physical exercise

## Abstract

**Simple Summary:**

The COVID-19 pandemic has been associated with increased mortality worldwide. Cancer patients are among those at enhanced risk while already suffering from decreased quality of life (QoL) due to their disease. In the present study, we investigated QoL in 100 brain tumor patients and relatives across a twelve-week timespan during the first COVID-related lockdown (04–07/2020) in detail. Compared to the general population, both patients and relatives showed significant distress, anxiety, and depression, with patients more at risk. QoL within a family—between patients and relatives—was correlated. While QoL did not change over time, acceptance of lockdown measures decreased towards the end of the study period. Finally, QoL was strongly associated with the number of weekly social contacts. These findings shed light on the psychosocial situation of a vulnerable cancer population during the COVID pandemic and indicate the need for targeted psychosocial interventions in these patients and their relatives.

**Abstract:**

The COVID-19 pandemic is associated with significant morbidity, mortality, and restrictions on everyday life worldwide. This may be especially challenging for brain tumor patients given increased vulnerability due to their pre-existing condition. Here, we aimed to investigate the quality of life (QoL) in brain tumor patients and relatives in this setting. Over twelve weeks during the first wave of the pandemic (04–07/2020), brain tumor patients and their families from two large German tertiary care centers were asked to complete weekly questionnaires for anxiety, depression, distress, and well-being. Information regarding social support and living conditions was also collected. One hundred participants (63 patients, 37 relatives) completed 729 questionnaires over the course of the study. Compared to relatives, patients showed more depressive symptoms (*p* < 0.001) and reduced well-being (*p* = 0.013). While acceptance of lockdown measures decreased over time, QoL remained stable. QoL measures between patients and their families were weakly or moderately correlated. The number of social contacts was strongly associated with QoL. Age, living conditions, ongoing therapy, employment, and physical activity were other predictors. QoL is correlated between patients and their families and heavily depends on social support factors, indicating the need to focus on the entire family and their social situation for QoL interventions during the pandemic.

## 1. Introduction

The ongoing COVID-19 pandemic has been associated with increased morbidity and mortality worldwide. To efficiently combat and manage the spread of the disease, significant restrictions of everyday life have been implemented, prompting discussion regarding associated mental health challenges [1]. First studies have demonstrated that the ongoing pandemic and its ramifications have indeed been associated with an increase in depressive symptoms in the wider population [2,3]. Calls for an increase in mental health interventions have subsequently followed [4,5].

In this setting, tumor patients have been shown to be a group especially at risk [6], both for worse outcomes when infected with the virus [7,8] as well as an exacerbation of depressive symptoms given their already-vulnerable state [9]. Among cancer patients, brain tumor patients have long been identified to be among those especially at risk for mental health challenges [10,11]. Relatives of brain tumor patients are similarly known to carry a significant psychosocial burden [12]. Thus, a closer look is needed to describe the difficulties faced by brain tumor patients and their families during the pandemic to better understand and, possibly, ameliorate the challenges they face.

The present longitudinal, bi-institutional study was designed to assess the mental health status of brain tumor patients during the first lockdown phase of the pandemic. Besides, we also set out to investigate the situation immediate relatives face. Finally, we aimed to identify factors associated with mental health in an effort to possibly help guide quality of life (QoL)-based interventions.

## 2. Materials and Methods

This exploratory longitudinal cohort observational study was approved by the Ethics Committee of the Westfalian Wilhelms-University Münster and the Medical Association Westphalia-Lippe (No. 2020-269-f-S). All participants consented to the study in writing. The study protocol was prepared in accordance with the principles outlined in the Helsinki declaration.

Participants, including brain tumor patients and immediate relatives, received weekly questionnaires over 12 weeks. The study period was between 22 April and 15 July 2020. Questionnaires were administered online using the soscisurvey tool (https://www.soscisurvey.de/) (accessed regularly between April and July 2020) and always addressed the prior seven-day timeframe. Links were sent to participants every Wednesday morning. A reminder was sent to all participants 2 days later, regardless of completion of the questionnaire in the meantime.

To allow for longitudinal follow-up, participants were asked to design an individual code, to be used every week. A box allowed indicating their status as patient or relative.

### 2.1. Questionnaire Instruments

The same questionnaire was administered to patients and relatives with disease characteristics to be filled out for brain tumor patients only. It contained 5 Sections:

#### 2.1.1. Demographic and Disease Characteristics

Age, sex, height, weight, and relationship status were included. Patient diagnosis, including most recent neuropathology-determined World Health Organization (WHO) grading at the time of study participation (for the respective tumor entity) and ongoing therapy (to be answered affirmatively if within two weeks of surgical intervention, or under ongoing radiation therapy, chemotherapy, or immunotherapy) were also collected.

#### 2.1.2. Living Conditions

Here, apartment vs. house, size of living area (<100 m^2^ vs. >100 m^2^), outdoor facilities at home (e.g., garden), and whether participants were living alone were collected.

#### 2.1.3. Personal Behavior

We inquired about the number of weekly contacts to friends, acquaintances, or family outside the home environment. We asked to include any contact independent of nature (in-person, via telephone, via video tools). Categories included 0–3, 4–6, 7–10, and more than 10 per week. We also inquired about the presence of a day job. Finally, the frequency of physical exercise (<1/week vs. 1+/week) was collected.

#### 2.1.4. Isolation Questionnaire

The questionnaire ISOLA (short for isolation) was taken from a previous study upon approval by that study’s lead author [13]. It was translated into German by a certified translator and slightly adapted to reflect the difference in the situation (the questionnaire was originally designed to assess isolation after stem cell transplantation). No additive score was built, and each measure was individually analyzed. The original questionnaire as well as its German translation can be found in Table S1.

#### 2.1.5. QoL Outcome Measures

We collected four quality of life measures:

Anxiety and depression were measured by the Hospital Anxiety and Depression Scale (HADS) questionnaires. Briefly, 7 individual items for each outcome graded from 0 to 3 are added to a total score of 0 to 21. High scores are known to correlate with a high symptom burden. The use of this measure is common in cancer patients [14].

The distress thermometer is a simple one-item scale that allows participants to grade individual levels of distress from 0 to 10. Ten marks the highest level of distress. Its use is recommended in clinical practice [15].

Finally, well-being was assessed using the WHO5 well-being score. It includes five statements on quality of life graded from 0 to 5. Scores are obtained by building the sum of all values ranging from 0 to 25 and transforming this sum to reflect a range from 0% (0 points) to 100% (25 points). Here, high scores are associated with well-being. The score has found ample use across different fields, including in cancer patients [16].

### 2.2. Study Population and Recruitment

Participants were adult brain tumor patients and immediate adult relatives of brain tumor patients (partners, spouses, parents, adult children, or siblings). All brain tumor patients had undergone surgical evaluation during their course of treatment. As per the standard of care in both centers, patients were routinely approached regarding their interest to be included on an email list focusing on psychosocial support opportunities. In this setting, mailing lists designed by the psycho-oncologic services at both Münster University Hospital (MUE) and Bochum University Hospital (BOC) were used for recruitment. Participants younger than 18 years and those not consenting to participation were not included. However, no participants from either category asked to take part in this study. Thus, no potential participants had to be actively excluded from participation.

### 2.3. Statistics

The Stata software package (version 13.0; StataCorp, College Station, TX, USA) was used for data analysis with the alpha level set at 0.05. We used descriptive statistics to estimate frequencies, median with interquartile range (IQR) or mean with standard deviation (SD). Differences between patients and relatives or between center participants were assessed with two-tailed chi-square tests for categoric variables, Student’s t-tests for continuous variables demonstrating approximate normality as analyzed with the Shapiro Wilk test, and the Mann–Whitney U test for continuous variables not normally distributed. Relationships between the four outcomes were assessed using Spearman’s rho with the accompanying *p* value also presented. Associations between patient and family-reported outcomes were reported similarly. Relationship strength was interpreted based on previous studies [17,18]. Time-dependency of variables was assessed using visual plotting as well as testing the first two weeks vs. the last two weeks of participation using the tests outlined above. For time-dependency analyses we included all participants who participated at any timepoints, not limiting inclusion to only those participants who completed all 12 weeks of follow-up. Including all available data at a timepoint is routinely done in longitudinal settings [19,20,21,22]. Univariable and multivariable modeling was performed using all questionnaires available. Correlations were assessed using ordinal logistic regressions, as recommended previously [23], as all four outcome measures were ordinal variables. After univariable modeling, all parameters that showed statistically significant associations with at least two of the outcome variables were included for multivariable modeling. The resulting model was then calculated for all four outcomes.

## 3. Results

### 3.1. Patient Characteristics

**Figure 1:**
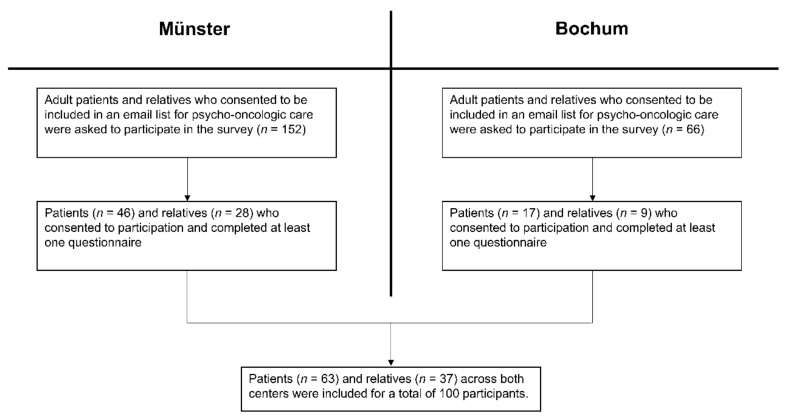
Participant inclusion, visualized for both centers.

One hundred adults (63 patients, 37 relatives) participated in the study after 218 patients and relatives had been contacted via email lists (Figure 1).

The patient cohort consisted of equal numbers of high- and low-grade brain tumor patients. Most patients had significant treatment experience: 99% had undergone surgery at least once and more than half of patients had received a second surgical procedure. A total of 81% of patients had undergone radiotherapy and 73% had previously been treated with chemotherapy, mostly with temozolomide. While no patients died during the study, 4 glioblastoma patients died between study completion and 28 February 2021 (Table S2). Given that the total number of patients currently under therapy was 20, we refrained from analyzing subgroups or testing different treatment paradigms for associations with quality of life given the high likelihood of underpowering.

Participants completed a median of 9 out of 12 weekly questionnaires distributed to them (interquartile range 2–11) for a total of 729 questionnaires. This results in a participation rate of 60.8% (729/1200) with 64.0% for patients (484/756) and 55.2% (245/444) for relatives. Overall, among included participants, patients were somewhat more likely to participate consistently than relatives (*p* = 0.002).

Patients were more often male, while relatives skewed female (*p* = 0.001, Table 1). Participating relatives were more likely to indicate larger living space (*p* = 0.033) and tended to work a day job more often (*p* = 0.055) compared to patients. Between all data points, relatives were also more likely to perform physical exercise at least once a week (*p* = 0.001). Between the four outcomes, patients demonstrated a higher HADS-Depression score across all timepoints (*p* < 0.001) and a lower WHO5 score (*p* = 0.013), indicating increased depressive symptom burden and reduced well-being when compared to relatives. When applying the commonly used cutoff of 10 for elevated depression values, 113/484 patient-submitted questionnaires (23.3%) but only 24/245 (9.8%) of relative-submitted questionnaires met the threshold (*p* < 0.001). Similar results were seen for anxiety (114/484 (23.5%) vs. 40/245 (16.3%), *p* = 0.024).

Some differences were apparent between centers (Table S3). In BOC, participants were less likely to live in a house (vs. living in an apartment, *p* < 0.001) and less likely to own outside facilities such as a garden (*p* = 0.011). High-grade tumors were somewhat more prevalent when compared to MUE (*p* = 0.023). Notably, only six patients with grade I brain tumors participated in total. Finally, weekly physical exercise was more often seen among patients from MUE (*p* < 0.001).

Moderate to strong correlations were seen between the four outcome variables, with positive associations between depression, anxiety, and distress scores and negative associations between these three items and well-being (Table S4).

### 3.2. Intra-Family Correlations

**Figure 2:**
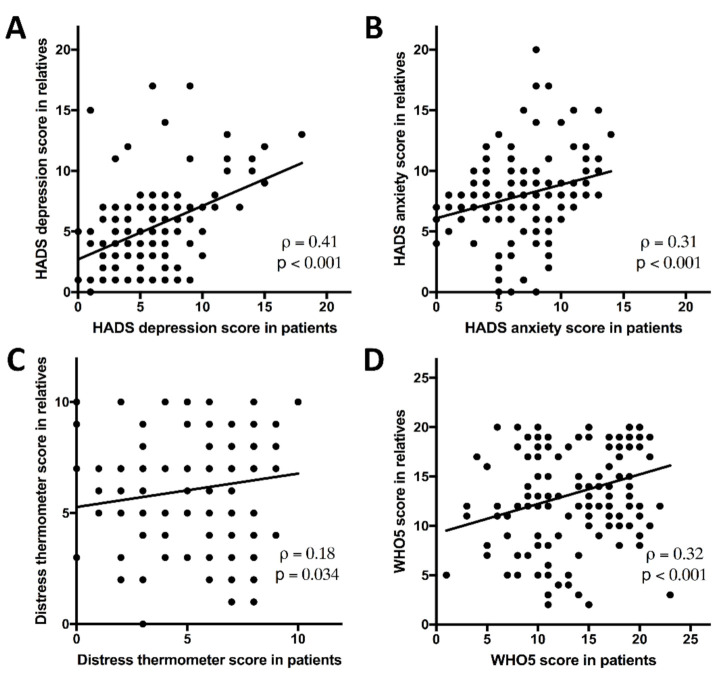
Patient scores and those obtained from their immediate relative in the same week are plotted on the x and y axis, respectively. All four outcomes, depression (**A**) and anxiety (**B**) as measured by the Hospital Anxiety and Depression Scale (HADS) score, distress thermometer (**C**), and WHO5 well-being score (**D**) demonstrate significant correlations between patient and relative-reported outcomes from the same family at the same timepoint. Spearman’s ρ and corresponding *p* value are given for each plot.

To evaluate influences between patients and their families, we matched a patient’s QoL data with QoL outcomes indicated by his or her relative within the same week. We thus generated a plot graph (Figure 2). Spearman’s correlation demonstrated weak to moderate associations that met levels of significance for all four outcomes.

### 3.3. Changes over Time

**Figure 3:**
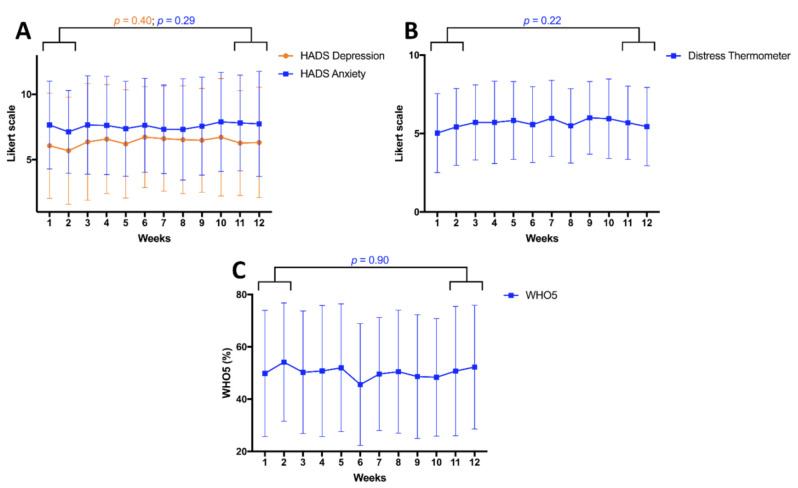
There were no changes over time for the four main outcomes of depressive symptoms and anxiety measured using the Hospital Anxiety and Depression Score (HADS); (**A**), the distress thermometer (**B**), and the WHO5 well-being score (**C**). Results are demonstrated for all participants (n = 100) and any participant who completed a questionnaire at the relevant timepoint was included. A graph depicting results for patients and relatives separately can be found in Figure S1.

We then assessed changes in QoL occurring across the twelve-week period. Interestingly, QoL appeared to remain stable and no changes were seen for the four main outcome parameters (Figure 3), even when substratified by patients and relatives (Supplementary Figure S1). Analyses included any participant who completed the survey in the specific week, resulting in slight changes in the study population between weeks. However, participation was largely stable over time (median: 59; interquartile range: 57–64; Table S5).

**Figure 4:**
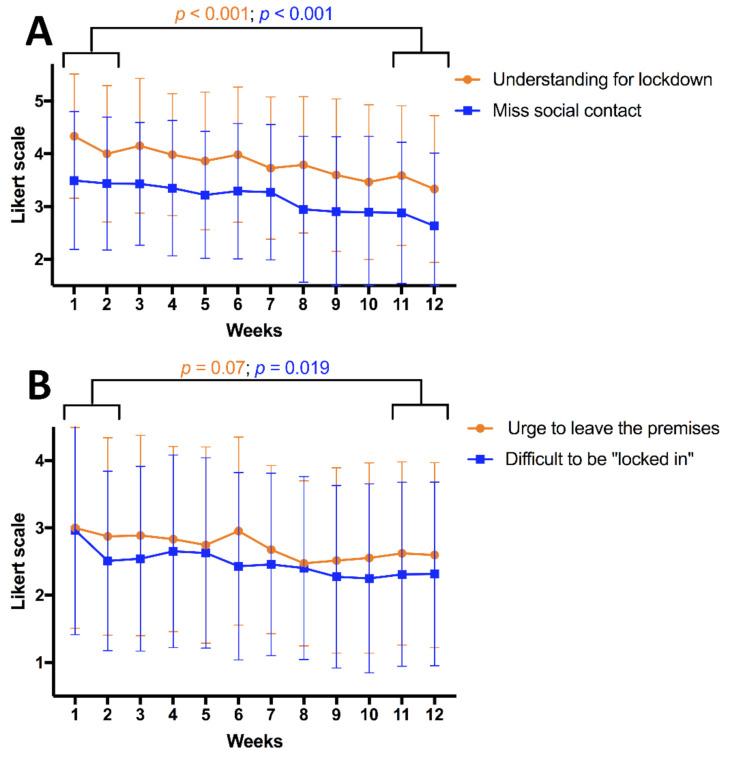
Changes in perception of the isolation over the course of the twelve-week study period. Answers correspond to items 2 and 3 (**A**) and 17 and 5 (**B**) from the ISOLA questionnaire, respectively. Data are presented as mean and standard deviation. Statistical comparisons were made between the first and last two weeks using the Mann–Whitney *U* test. Results are demonstrated for all participants (*n* = 100) and any participant who completed a questionnaire at the relevant timepoint was included. A graph depicting results for patients and relatives separately can be found in Figure S2.

However, over time, participants described fewer emotional difficulties with being under lockdown (*p* = 0.019, last two vs. first two weeks). Similarly, they tended to miss social contacts less at the conclusion of the study period when compared to the beginning (*p* < 0.001). Finally, the urge to leave the house or apartment also appeared to be decreased (*p* = 0.07). In parallel, however, understanding regarding the lockdown conditions decreased among participants (*p* < 0.001, Figure 4). Again, no significant differences regarding these trends were apparent between patients and relatives (Figure S2).

To better understand our longitudinal results against the backdrop of the COVID-19 pandemic, we obtained epidemiologic data regarding total cases, daily new infections, and total deaths during our study period from the Robert-Koch-Institut’s website (the Robert-Koch-Institut is responsible for maintaining epidemiologic registries during the pandemic). Results are presented in Supplementary Figure S3 with numbers for Germany and the region of North Rhine-Westphalia, where both study institutions are located. Data demonstrate a constant increase of cases during the study period, but with a decelerating trend.

Prior to the study period and in effect for the entire time, German authorities mandated physical distancing measures. Meetings were only allowed with a single member of another household and only outside and under observance of a minimum distance of 2 m. Larger gatherings were prohibited. Most businesses requiring physical presence were closed except for the medical field and grocery stores. Citizens were asked to stay at home and minimize social contact. A global travel warning was also in effect that was only lifted for European Union countries on 3 June 2020. Schools were slowly reopened at the end of April 2020, initially only for final-year students.

### 3.4. Influencing Factors

Finally, we aimed to identify parameters influencing QoL. For this analysis, we used all available questionnaires and first performed univariable analyses assessing associations with the personal details collected (Table 2). Generally, participants who were patients (vs. relatives), of older age, less physically active, with no day job, no outside facilities in their living premises, or who were under ongoing therapy had worse QoL. However, the most consistent predictor of QoL was the number of social contacts as odds ratios indicated that quality of life strongly improved with increased social contacts. There was no difference in QoL between low-grade and high-grade brain tumors.

To evaluate these findings in a multivariable setting, we chose to include all parameters that were significantly associated with at least two of the outcomes in a single model (Table 3). We thus included sex, patients vs. relatives, age, physical exercise frequency, outdoor facility at living premises, day job, relationship status, apartment vs. house, and ongoing therapy. All four outcomes were then assessed using 723 complete datasets. Patient status, higher age, less physical exercise, lack of outdoor facilities, no job, and ongoing therapy were again associated with worse QoL. Across all four outcomes, the number of social contacts again was a strong determinant of quality of life.

## 4. Discussion

The present bi-institutional study was designed to assess the mental health of brain tumor patients and their family during the ongoing pandemic, shed light on longitudinal changes during the first wave of the pandemic/first lockdown phase in early summer 2020, and finally identify factors associated with quality of life in brain tumor patients.

In the study, we included 100 participants, a majority of whom were patients. There are several plausible reasons for patients being more likely to participate in the study and also being more likely to consistently complete weekly questionnaires. First, patients were more directly involved in their care at the centers and, thus, the study recruitment. Second, there is a lower likelihood that patients hold a day job (as also evidenced in our study population as a trend) possibly resulting in more time to participate. Third, the fact that we limited participants to adults, precluding minors (e.g., patients’ children) from participating, may have limited the number of relatives any patient could involve in the study. In nearly all cases, only a single relative was involved in the study per patient.

Participants were recruited in two Western German tertiary care centers. This is reflected in the trimodal treatment most patients had already undergone prior to study participation. The extent of treatment is not unusual for brain tumor patients and may help define them as a specific cohort when compared to relatives or the general population, resulting in distinct QoL results discussed below. Within the study cohort, there were few major differences between the two centers: Participants in Bochum, a city within the heavily populated industrialized Ruhr area, tended to live in apartments more and were less likely to have outdoor facilities, whereas the contrary was true for participants from Münster, a city located in the more rural northwestern part of Germany. Physical activity was increased in Münster, possibly a result of the individualized brain tumor exercise program offered there, but not in Bochum. Exercise was also more common in relatives, as patients are often discouraged from or skeptical towards exercise given their disease [24].

### 4.1. Mental Health Challenges for Patients during the COVID Pandemic

Roughly 23% of examined patient questionnaires met the criteria for elevated depressive symptom load. Far fewer relatives were similarly burdened. This points to a high patient-specific depressive symptom burden and is a 5–10% higher ratio than pre-COVID brain tumor patient studies found [25,26,27,28]. Interestingly, the same is not true for anxiety, where our findings match the range defined by these previous studies [28,29,30]. Independent from this, absolute HADS values continue to be widely elevated relative to the general population [14] and point to a need for broad psychooncological screening among brain tumor patients.

For distress, a pooled pre-COVID-19 study including more than 2000 brain tumor patients found that distress was 4 points or higher in only 41% [11], a strong difference from our study where the median value among patients is 6. However, individual studies have reported median distress values similar to ours [25,31]. These values indicate significant suffering among brain tumor patients since most studies indicate a cutoff of 6 points for interventions [31], which is the median distress we found in our patients.

Finally, well-being was decreased by about 10% compared to pre-intervention levels of a previous QoL study at one of our centers [29].

Combined, these findings suggest only slightly enhanced depressive symptoms and, possibly, distress in brain tumor patients during the pandemic compared to before. More importantly, the median values we found indicate the need for wide-spread QoL screening among brain tumor patients as a significant part of the study population met the criteria for needing QoL interventions.

Generally, quality of life measures have also been demonstrated to be associated with overall survival (OS) [32]. While our study was not designed for survival analyses (and only four patients died in the roughly 6 months following study participation, precluding statistical analyses), survival analyses should be considered when designing future large-scale COVID quality of life studies.

### 4.2. Findings on Relatives’ Quality of Life

Recently, an increasing number of brain tumor studies have focused on relatives’ QoL as they have been shown to be significantly distressed as well [12,33]. Our results point to a similar degree of symptoms of anxiety and distress between patients and relatives, somewhat deviating from the single different QoL study in brain tumor patients during the COVID pandemic: Voisin et al., in an online-based one-timepoint-study, reported findings of increased anxiety among relatives [30]. Here, regional variation may play a role as less than 10% of Voisin et al.’s population emanate from continental Europe. Second, recruitment at our study was performed directly at the medical centers, while Voisin et al. relied on brain tumor charities to spread the study, possibly resulting in different response demographics.

Interestingly, we were able to show that there is a direct correlation when pairing patient’s QoL to their family’s QoL during the same time during the pandemic. Correlations, while highly significant, were of weak to moderate strength, likely due to the small participant cohort and, in the case of the distress thermometer, the lack of granularity of the outcome measure. Nonetheless, these findings underline that patients’ well-being has immediate ramifications for their families. It thus emphasizes the need to focus on both the patient *and* his social surroundings when preparing quality of life interventions. This mirrors a previous report on breast and colon cancer patients [34].

### 4.3. Changes over Time

Several studies report a worsening of QoL in the general population during the lockdown [35,36,37]. This was not the case in our study where all QoL indicators remained largely stable over a twelve-week period. This is in line with Voisin et al.’s previous brain tumor COVID study, which—while only investigating a single timepoint—found more than 95% of participants indicating they coped “fairly well” or better with the pandemic [30]. The findings are also supported by previous studies indicating distressed communities do not experience a worsening of symptoms during the pandemic while exhibiting continuously high levels of depression and anxiety [38,39]. This result is relevant as the COVID-19 infection numbers continuously increased and the lockdown, originally mandated on 23 March, was repeatedly prolonged during the study period. Given the extent of the measures the lockdown likely significantly affected our participants. Nonetheless, while the lockdown certainly influenced our study parameters, patients and their relatives seem equipped to handle significant long-running everyday restrictions on their social life with no major QoL deterioration over time.

Interestingly, some changes were seen over the twelve-week period: participants indicated lower desire to leave their house/apartment and missed social contacts less as time progressed. Similar to a previous study, this points to an adaptation to lockdown rules [40]. Another strong change over time was a decrease in acceptance of lockdown measures. This indicates that while brain tumor patients and their relatives represent a risk cohort, they still respond similarly to lockdown measures when compared to the overall population: a public policy study shows that acceptance for lockdowns among the general population is heavily dependent on and inversely correlated with their length [41].

Finally, it is difficult to determine any effect of the specific number of COVID-19 cases locally on psychosocial health over time. During our study, case numbers locally and nationally continuously increased, but with a decelerating trend. This may have helped stabilize QoL in our study population. It may have also played a role that, while significantly challenged, the German healthcare system was at no time overwhelmed by the pandemic. In any case, the absence of major escalations (regarding government regulations or disease spread) during the study period likely contributed to the stable QoL findings and loss of acceptance for the lockdown.

### 4.4. Social Support Is a Strong Predictor of Quality of Life

In our univariable analyses, social support factors were strongly associated with all QoL measures. These findings were supported in our multivariable model where the number of social contacts relevantly predicted all outcomes. QoL consistently rose with an increased number of contacts. Ten or more social contacts were associated with a 70% reduced risk for more depression symptoms, a 39% reduced risk for more anxiety symptoms, and a 65% reduced risk for increased distress while enhancing the chance of increased well-being by 73% when compared to 0–3 social contacts/week. This potentially poses some key challenges regarding lockdown adherence, social distancing, and mental health. Unfortunately, in our study, we did not differentiate between in-person and phone/video contacts, limiting conclusions regarding the effect of in-person vs. physically distant contacts.

These findings, which mirror studies in the general public [42,43], inform the need to make technology available to all brain tumor patients to enable physically distant social contacts. Low-income households and the elderly are especially disadvantaged here [44] with the latter experiencing decreased QoL in our study, making the need more pressing.

People out of work [45] and without a partner [46] were similarly at risk in our study, as in others as well. Outdoor facilities are also beneficial, as has been hypothesized [47]. Females tended to express more anxiety, another similarity between brain tumor patients in our study and the general population [45]. While participants in apartments had better QoL in our study, this likely relates to this cohort being significantly younger on average compared to participants living in houses. Ongoing therapy was associated with increased anxiety and distress, although we were unable to test treatment subgroups due to underpowering. The effects we saw for the patients under therapy may be related to the respective therapy itself. However, treatment-related QoL differences in randomized treatment studies are scarce [48]. Alternatively, the exposure to hospital environments during a pandemic, a point of some contention in the brain tumor care provider community [49], may play a role. Additionally, “ongoing therapy” may be indicative of tumor progression. A previous study hypothesized that tumor progression may indeed be a more relevant factor than tumor grade [50], a finding mirrored in our study as tumor grade was not relevantly correlated with QoL. Other studies have similarly reported that tumor grade is not a relevant factor for QoL [51,52,53]. However, all studies, including this investigation, may not have had adequate patient numbers to identify any differences by tumor grade. In any case, if existent, tumor grade-related QoL effects are likely small, underlining the key importance of social characteristics in comparison.

There are some limitations to this study. First, we included only one hundred participants from two neighboring Western German centers, with numbers and regional proximity possibly limiting generalizability. Similarly, subgroup analyses based on clinical data and treatment regimens were not feasible. Second, as discussed above, when collecting the number of social contacts per week we did not collect the nature (in-person vs. physically distant), somewhat limiting conclusions for lockdown consequences and mental health. Third, as always with self-reported outcomes from volunteer participants, selection and response biases apply.

## 5. Conclusions

During the first wave of the COVID-19 pandemic in spring 2020 and its associated lockdown measures, both patients and relatives exhibited significant levels of depression, anxiety, and distress, with patients more at risk. Quality of life between patients and their families is correlated, informing the need to focus on the entire family for mental health interventions during the pandemic. Especially during the lockdown, social contacts and support remain key determinants for quality of life.

## Figures and Tables

**Table 1:** Participant characteristics by status. Data are summarized as number and frequency, mean and standard deviation, or median and interquartile range, as appropriate.

**All Participants**	***n***	**All (*n* = 100)**	**Patients (*n* = 63)**	**Relatives (*n* = 37)**	***p***
Age, years, mean (SD)	100	48.2 (13.2)	48.3 (12.2)	48.1 (14.9)	0.95 ^+^
Center	100				0.77 ^#^
Münster, *n* (%)		74 (74.0)	46 (73.0)	28 (75.7)	
Bochum, *n* (%)		26 (26.0)	17 (27.0)	9 (24.3)	
Sex	100				0.001 ^#^
Male, *n* (%)		44 (44.0)	36 (57.1)	8 (21.6)	
Female, *n* (%)		56 (56.0)	27 (42.9)	29 (78.4)	
Number of questionnaires per participant, median (IQR)	100	9 (2–11)	10 (2–11)	8 (2–11)	0.13 ^x^
BMI, kg/m^2^ (IQR)	100	24.5 (20.7–27.0)	24.5 (21.3–27.2)	23.6 (20.5–26.9)	0.29 ^x^
Living facilities with outdoor area	97				0.58 ^#^
No, *n* (%)		19 (19.6)	13 (21.3)	6 (16.7)	
Yes, *n* (%)		78 (80.4)	48 (78.7)	30 (83.3)	
Relationship status	98				0.06 ^#^
Single, *n* (%)		14 (14.3)	12 (16.7)	2 (5.6)	
In a relationship, *n* (%)		84 (85.7)	50 (83.3)	34 (94.4)	
Flatmates	100				0.62 ^#^
No, *n* (%)		13 (13.0)	9 (14.3)	4 (10.8)	
Yes, *n* (%)		87 (87.0)	54 (85.7)	33 (89.2)	
Day job	96				0.067 ^#^
No, *n* (%)		38 (39.6)	28 (46.7)	10 (27.8)	
Yes, *n* (%)		58 (60.4)	32 (53.3)	26 (72.2)	
House vs. Apartment	97				0.11 ^#^
House, *n* (%)		66 (68.0)	38 (62.3)	28 (77.8)	
Apartment, *n* (%)		31 (32.0)	23 (37.7)	9 (23.2)	
Area	97				**0.041 ^#^**
<100 m^2^, *n* (%)		37 (38.1)	28 (45.9)	9 (25.0)	
>100 m^2^, *n* (%)		60 (61.9)	33 (54.1)	27 (75.0)	
Diagnosis of the patient within the family	100				0.90 ^#^
Meningeoma, *n* (%)		7 (7.0)	5 (7.9)	2 (5.4)	
Astrocytoma, *n* (%)		35 (35.0)	23 (36.5)	12 (32.4)	
GBM, *n* (%)		31 (31.0)	20 (31.7)	11 (29.7)	
Oligodendroglioma, *n* (%)		15 (15.0)	8 (12.7)	7 (18.9)	
Others *, *n* (%)		12 (12.0)	7 (11.3)	5 (13.5)	
WHO brain tumor grading ^§^	100				0.84 ^#^
Low grade (WHO I + II), *n* (%)		50 (50.0)	32 (50.8)	18 (48.7)	
High grade (WHO III + IV), *n* (%)		50 (50.0)	31 (49.2)	19 (51.4)	
Ongoing therapy	100				0.94 ^#^
No, *n* (%)		68 (68.0)	43 (68.3)	25 (67.6)	
Yes, *n* (%)		32 (33.0)	20 (31.7)	12 (32.4)	
**All Questionnaires**	***n***	**All (*n* = 729)**	**Patients (*n* = 484)**	**Relatives (*n* = 245)**	***p***
Physical exercise frequency	729				**<0.001 ^#^**
Occasionally (<1/week), *n* (%)		179 (24.6)	138 (28.5)	41 (16.7)	
Often (≥1/week), *n* (%)		550 (75.5)	346 (71.5)	204 (83.3)	
Social contacts/week	729				**0.74 ^#^**
0–3, *n* (%)		227 (31.1)	157 (32.4)	70 (28.6)	
4–6, *n* (%)		186 (25.5)	120 (24.8)	66 (26.9)	
7–10, *n* (%)		136 (18.7)	88 (18.2)	48 (19.6)	
10+, *n* (%)		180 (24.7)	119 (24.6)	61 (24.9)	
HADS-Depression, median (IQR)	729	6 (3–9)	7 (3–10)	6 (3–7)	**<0.001 ^x^**
HADS Anxiety, median (IQR)	729	8 (5–10)	8 (5–10)	8 (6–10)	**0.99 ^x^**
Distress Thermometer, median (IQR)	729	6 (4–8)	6 (3–8)	6 (4–8)	**0.19 ^x^**
WHO5, median (IQR)	729	48 (32–72)	48 (28–72)	52 (36–72)	**0.013 ^x^**

SD: standard deviation; IQR: interquartile range; BMI: body mass index; WHO: World Health Organization; HADS: Hospital Anxiety and Depression Scale. * Other tumors include neurinoma (*n* = 3), plexus papilloma (*n* = 3), ependymoma (*n* = 2), solitary fibrous tumor (*n* = 2), germinoma (*n* = 1), ganglioglioma (*n* = 1); ^§^ most recent grading as determined by a neuropathologist; ^+^ *t* test; ^#^ χ^2^ test; ^x^ Mann–Whitney *U* test.

**Table 2:** Univariable ordinal logistic regression analyses correlating depressive symptoms and anxiety as measured by the Hospital Anxiety and Depression Scale (HADS), distress as measured by the distress thermometer, and well-being as quantified by the WHO5 scale with participant characteristics. Odds ratios, 95% confidence intervals, and *p* values are given for each parameter.

Univariable Analyses		HADS Depression (Scale 0–21, Per Point)		HADS Anxiety (Scale 0–21, Per Point)		Distress Thermometer (Scale 0–10, Per Point)		WHO5 (Scale 0–100%, Per 4%)	
Parameter	*n*	OR (95% CI)	*p*	OR (95% CI)	*p*	OR (95% CI)	*p*	OR (95% CI)	*p*
Sex	729								
Female		1.00	**Ref.**	**1.00**	**Ref.**	**1.00**	**Ref.**	**1.00**	**Ref.**
Male		1.53 (1.18–1.98)	**0.001**	**0.8 (0.62–1.04)**	**0.099**	**1.01 (0.78–1.31)**	**0.92**	**0.95 (0.73–1.23)**	**0.69**
Role	729								
Relatives		1.00	**Ref.**	**1.00**	**Ref.**	**1.00**	**Ref.**	**1.00**	**Ref.**
Patients		1.58 (1.21–2.06)	**0.001**	**1 (0.77–1.3)**	**0.99**	**0.84 (0.64–1.09)**	**0.19**	**0.72 (0.55–0.93)**	**0.014**
Center	729								
Münster		1.00	**Ref.**	**1.00**	**Ref.**	**1.00**	**Ref.**	**1.00**	**Ref.**
Bochum		0.81 (0.61–1.08)	**0.15**	**0.81 (0.61–1.08)**	**0.16**	**1.01 (0.75–1.35)**	**0.96**	**1 (0.75–1.33)**	**1.00**
Age	729	1.05 (1.04–1.06)	**<0.001**	**1.03 (1.02–1.04)**	**<0.001**	**1.01 (1–1.02)**	**0.12**	**0.97 (0.96–0.98)**	**<0.001**
BMI	729	1.05 (1.02–1.08)	**0.001**	**0.99 (0.96–1.01)**	**0.33**	**0.98 (0.95–1.01)**	**0.13**	**1.01 (0.99–1.04)**	**0.36**
Physical exercise frequency	729								
Occasionally (<1/week)		1.00	**Ref.**	**1.00**	**Ref.**	**1.00**	**Ref.**	**1.00**	**Ref.**
Often (≥1/week)		0.6 (0.45–0.8)	**0.001**	**1.05 (0.78–1.42)**	**0.74**	**1.11 (0.83–1.48)**	**0.49**	**1.77 (1.32–2.38)**	**<0.001**
Living facilities with outdoor area	726								
No		1.00	**Ref.**	**1.00**	**Ref.**	**1.00**	**Ref.**	**1.00**	**Ref.**
Yes		0.57 (0.41–0.8)	**0.001**	**0.9 (0.66–1.22)**	**0.49**	**0.98 (0.71–1.35)**	**0.88**	**1.96 (1.43–2.69)**	**<0.001**
Relationship status	724								
Single		1.00	**Ref.**	**1.00**	**Ref.**	**1.00**	**Ref.**	**1.00**	**Ref.**
In a relationship		0.76 (0.54–1.06)	**0.11**	**1.04 (0.74–1.46)**	**0.82**	**0.98 (0.7–1.36)**	**0.88**	**1.55 (1.11–2.17)**	**0.011**
Flatmates	729								
No		1.00	**Ref.**	**1.00**	**Ref.**	**1.00**	**Ref.**	**1.00**	**Ref.**
Yes		0.82 (0.57–1.16)	**0.25**	**1.11 (0.78–1.59)**	**0.56**	**1.17 (0.83–1.65)**	**0.37**	**0.77 (0.54–1.09)**	**0.14**
Social contacts/week	729								
0–3		1.00	**Ref.**	**1.00**	**Ref.**	**1.00**	**Ref.**	**1.00**	**Ref.**
4–6		0.70 (0.49–0.98)	**0.039**	**0.89 (0.64–1.25)**	**0.51**	**0.75 (0.53–1.05)**	**0.092**	**1.01 (0.72–1.41)**	**0.97**
7–10		0.52 (0.36–0.78)	**0.001**	**0.72 (0.5–1.05)**	**0.091**	**0.72 (0.5–1.05)**	**0.091**	**1.14 (0.79–1.66)**	**0.49**
10+		0.26 (0.18–0.36)	**<0.001**	**0.53 (0.38–0.75)**	**<0.001**	**0.38 (0.27–0.54)**	**<0.001**	**2.04 (1.44–2.9)**	**<0.001**
Day job	725								
No		1.00	**Ref.**	**1.00**	**Ref.**	**1.00**	**Ref.**	**1.00**	**Ref.**
Yes		0.55 (0.42–0.71)	**<0.001**	**0.58 (0.45–0.76)**	**<0.001**	**0.87 (0.67–1.12)**	**0.27**	**1.97 (1.52–2.55)**	**<0.001**
House vs. Apartment	726								
House		1.00	**Ref.**	**1.00**	**Ref.**	**1.00**	**Ref.**	**1.00**	**Ref.**
Apartment		0.74 (0.56–0.97)	**0.027**	**0.66 (0.50–0.86)**	**0.002**	**0.83 (0.63–1.09)**	**0.17**	**1 (0.76–1.31)**	**1.00**
Area	726								
<100 m^2^		1.00	**Ref.**	**1.00**	**Ref.**	**1.00**	**Ref.**	**1.00**	**Ref.**
>100 m^2^		1.13 (0.98–1.29)	**0.086**	**1.13 (0.99–1.29)**	**0.072**	**1.11 (0.97–1.26)**	**0.13**	**0.93 (0.82–1.06)**	**0.29**
WHO grade	729								
low grade (I + II)		1.00	**Ref.**	**1.00**	**Ref.**	**1.00**	**Ref.**	**1.00**	**Ref.**
high grade (III + IV)		0.9 (0.7–1.16)	**0.43**	**0.94 (0.73–1.22)**	**0.67**	**1.02 (0.79–1.33)**	**0.85**	**1.33 (1.03–1.71)**	**0.029**
Ongoing therapy	729								
No		1.00	**Ref.**	**1.00**	**Ref.**	**1.00**	**Ref.**	**1.00**	**Ref.**
Yes		1.02 (0.78–1.34)	**0.87**	**1.5 (1.14–1.97)**	**0.004**	**1.79 (1.36–2.35)**	**<0.001**	**0.74 (0.57–0.96)**	**0.025**

HADS: Hospital Anxiety and Depression Scale; WHO: World Health Organization; OR: odds ratio; CI: confidence interval; BMI: body mass index.

**Table 3:** Multivariable analyses correlating depressive symptoms and anxiety as measured by the Hospital Anxiety and Depression Scale (HADS), distress as measured by the distress thermometer, and well-being as quantified by the WHO5 scale with participant characteristics. Multivariable ordinal logistic regressions were performed to assess associations between parameters and outcomes. The same model was chosen for all four outcomes including all parameters that had shown associations with at least two outcomes in the univariable setting. A total of 723 questionnaires were included. Odds ratios, 95% confidence intervals, and *p* values are given for each parameter.

Multivariable Analyses	HADS Depression (Scale 0–25, Per Point)		HADS Anxiety (Scale 0–25, Per Point)		Distress Thermometer (Scale 0–10, Per Point)		WHO5 (Scale 0–100%, Per 4%)	
*n* = 723	OR (95% CI)	*p*	OR (95% CI)	*p*	OR (95% CI)	*p*	OR (95% CI)	*p*
Role								
Relatives	Ref. 1.00		Ref. 1.00		Ref. 1.00		Ref. 1.00	
Patients	1.51 (1.13–2)	**0.005**	1.05 (0.8–1.39)	0.719	0.82 (0.61–1.09)	0.169	0.87 (0.66–1.15)	0.343
Age	1.05 (1.04–1.07)	**<0.001**	1.03 (1.02–1.04)	**<0.001**	1.01 (0.99–1.02)	0.308	0.97 (0.96–0.98)	**<0.001**
Physical exercise frequency								
Occasionally (<1/week)	Ref. 1.00		Ref. 1.00		Ref. 1.00		Ref. 1.00	
Often (≥1/week)	0.73 (0.53–0.99)	**0.046**	1.11 (0.81–1.52)	0.52	1.12 (0.82–1.53)	0.466	1.63 (1.19–2.23)	**0.002**
Living facilities with outdoor area								
No	Ref. 1.00		Ref. 1.00		Ref. 1.00		Ref. 1.00	
Yes	0.17 (0.11–0.26)	**<0.001**	0.43 (0.28–0.65)	**<0.001**	0.72 (0.47–1.09)	0.119	3.3 (2.16–5.04)	**<0.001**
Social contacts/week								
0–3	Ref. 1.00		Ref. 1.00		Ref. 1.00		Ref. 1.00	
4–6	0.68 (0.48–0.95)	**0.026**	0.83 (0.59–1.16)	0.267	0.67 (0.48–0.95)	**0.026**	1.04 (0.74–1.46)	0.829
7–10	0.38 (0.25–0.56)	**<0.001**	0.64 (0.43–0.93)	**0.02**	0.65 (0.44–0.95)	**0.026**	1.39 (0.95–2.03)	0.088
10+	0.3 (0.21–0.43)	**<0.001**	0.61 (0.43–0.87)	**0.006**	0.35 (0.24–0.51)	**<0.001**	1.73 (1.2–2.49)	**0.003**
Day job								
No	Ref. 1.00		Ref. 1.00		Ref. 1.00		Ref. 1.00	
Yes	0.65 (0.5–0.86)	**0.002**	0.69 (0.52–0.92)	**0.01**	0.88 (0.67–1.16)	0.369	1.8 (1.36–2.38)	**<0.001**
Living facilities								
House	Ref. 1.00		Ref. 1.00		Ref. 1.00		Ref. 1.00	
Apartment	0.33 (0.23–0.47)	**<0.001**	0.55 (0.39–0.78)	**0.001**	0.86 (0.6–1.22)	0.393	1.52 (1.07–2.17)	**0.021**
Ongoing therapy								
No	Ref. 1.00		Ref. 1.00		Ref. 1.00		Ref. 1.00	
Yes	1.17 (0.88–1.56)	0.282	1.74 (1.3–2.32)	**<0.001**	1.95 (1.46–2.6)	**<0.001**	0.58 (0.43–0.77)	**<0.001**

HADS: Hospital Anxiety and Depression Scale; WHO: World Health Organization; OR: odds ratio; CI confidence interval; BMI: body mass index.

## Data Availability

All supporting data can be found in the manuscript.

## Supplementary Materials

The following are available online at https://www.mdpi.com/2072-6694/13/6/1276/s1, Table S1: Isolation questionnaire ISOLA with German translation, Table S2: Current and pre-treatment situation of the 63 patients included in the study, Table S3: Participant characteristics by center, Table S4: Correlation between outcome parameters among all 729 questionnaires, Table S5: Correlation between outcome parameters among all 729 questionnaires, Figure S1: Changes over time for outcomes, divided by patients and relatives, Figure S2: Changes in perception of the isolation over the course of the twelve-week study period, divided by patients and relatives, Figure S3: Development of COVID-19 total cases, daily change and COVID-related deaths.
