# Supplementary material for: Quality of Life in Brain Tumor Patients and Their Relatives Heavily Depends on Social Support Factors during the COVID-19 Pandemic
Source: Cancers (Basel). 2021 Mar 13;13(6):1276. doi: 10.3390/cancers13061276 (PMC7999211; doi:10.3390/cancers13061276)
Supplement: Supplementary file 1 [file cancers-13-01276-s001.pdf]

Table S1. Isolation questionnaire ISOLA as published by Biagoli et al. with German translation, as translated by a certified translation specialist. The questionnaire was slightly modified to reflect the ongoing pandemic (it was originally designed for patients under protective isolation after stem cell transplantation).

Item No.	English Version	German Version
1	I get bored because time passes slowly.	Mir wird langweilig, weil die Zeit langsam vergeht.
2	I understand why I have to stay in isolation.	Ich verstehe, warum ich in Isolation bleiben muss.
3	I miss contact with the outside world.	Ich vermisse den Kontakt mit der Außenwelt.
4	I can stay in the isolation room with serenity.	Ich kann voller Gelassenheit in Isolation in der Wohnung/im Haus bleiben.
5	Being shut inside a room is hard.	Innerhalb der Wohnung/des Hauses eingeschlossen zu sein, ist schwer.
6	It is a problem for me not being able to stay close to my loved ones.	Es ist ein Problem für mich, nicht in der Nähe meiner Lieben sein zu können.
7	I lack space to move around.	Mir fehlt der Platz, um mich zu bewegen.
8	Staying in isolation helps me to look at life from a new perspective.	Der Aufenthalt in der Isolation hilft mir, das Leben aus einer neuen Perspektive zu betrachten.
9	I need someone close to me to talk with.	Ich brauche jemanden, der mir nahesteht, um mit ihm zu sprechen.
10	Staying in isolation enhances my fears.	Der Aufenthalt in der Isolation verstärkt meine Ängste.
11	I prefer to stay on my own inside the room	Ich ziehe es vor, allein in der Wohnung/im Haus zu bleiben.
12	I feel cut off from the world.	Ich fühle mich von der Welt abgeschnitten.
13	Staying here on my own allows me to think more about myself.	Hier allein für mich zu sein, ermöglicht es mir, mehr über mich selbst nachzudenken.
14	I feel imprisoned.	Ich fühle mich wie im Gefängnis.
15	Staying in isolation makes me feel safe.	In der Isolation fühle ich mich sicher.
16	I feel detached from my loved ones	Ich fühle mich von meinen Lieben getrennt.
17	I feel I want to leave the room.	Ich habe das Gefühl, dass ich die Wohnung/das Haus verlassen möchte.

Table S2. Current and pre-treatment situation of the 63 patients included in the study.

	**Current Therapy**	
**All participants**	**n (%)**	**Comment**
Temozolomide	12 (19.0%)	2 patients in combination with CCNU,<br>2 in combination with radiation therapy,<br>1 in combination with hydroxyurea,<br>7 with no combination partner.
Radiation Therapy	4 (6.3%)	2 patients in combination with temozolomide,<br>1 with sequential PCV combination,<br>1 re-irradiation with no combination.
Bevacizumab	3 (5.1%)	1 patient with surgery in the same timeframe,<br>1 with irinotecan,<br>1 with no combination.
Regorafenibe	1 (1.6%)	1 patient with no combination.
Ifosfamide	1 (1.6%)	1 patient with no combination.
Surgery	1 (1.6%)	1 patient subsequent to bevacizumab .
	**Diagnosis & Prior Therapy**	
Year of Diagnosis		
Before 2015	22 (32.8%)	
2015-2017	13 (19.4%)	
2018	16 (23.9%)	
2019	10 (14.9%)	
2020	6 (9.0%)	
Surgery	66 (98.5%)	61 resections,<br>5 stereotactic biopsies.
Second surgery	36 (53.7%)	32 resections,<br>4 stereotactic biopsies.
Third surgery	11 (16.4%)	11 resections.
Radiation Therapy	54 (80.6%)	Mean radiation dose 56.9 Gy (standard deviation 3.0 Gy) in 1.8-2 Gy fractions.
Second radiation regimen	8 (11.9%)	1 stereotactic hypofractionated radiation therapy (5 x 5 Gy),<br>7 remaining: mean radiation dose: 39.1 Gy (standard deviation: 4.2 Gy) in 1.8-2 Gy fractions.

Any chemotherapy	49 (73.1%)	
Temozolomide-based chemotherapy	43 (64.2%)	
		**Follow-up**
Death during study	0 (0%)	
Death between study completion and February 28th, 2021	4 (6.0%)	All 4 patients had been diagnosed with a glioblastoma (WHO grade IV).

CCNU 1-(2-chloroethyl)-3-cyclohexyl-1-nitrosourea; PCV CCNU, procarbazine, and vincristine; Gy Gray; WHO World Health Organization.

Table S3. Participant characteristics by center. Data are summarized as number and frequency, mean and standard deviation or median and interquartile range, as appropriate.

	n	All (n = 100)	Münster (n = 74)	Bochum (n = 26)	*p*
Age, years, mean (SD)	100	48.2 (13.2)	48.4 (12.9)	47.6 (14.3)	0.80 +
Role	100				0.77 #
Patients, n (%)		63 (63.0)	46 (62.2)	17 (65.4)	
Relatives, n (%)		37 (37.0)	28 (37.8)	9 (34.6)	
Sex	100				0.84 #
Male, n (%)		44 (44.0)	33 (44.6)	11 (42.3)	
Female, n (%)		56 (56.0)	41 (55.4)	15 (57.7)	
Number of questionnaires per participant, median (IQR)	100	9 (2–11)	9.5 (2–11)	8 (3–10)	0.24 x
BMI, kg/m2	100	24.5 (20.7–27.0)	24.2 (20.5–26.6)	24.7 (22.0–29.0)	0.30 x
Living facilities with outdoor area	97				**0.011** #
No, n (%)		19 (19.6)	10 (13.7)	9 (37.5)	
Yes, n (%)		78 (80.4)	63 (86.3)	15 (62.5)	
Relationship status	98				0.70 #
Single, n (%)		14 (14.3)	10 (13.5)	4 (16.7)	
In a relationship, n (%)		84 (85.7)	64 (86.5)	20 (83.3)	
Flatmates	100				0.67 #
No, n (%)		13 (13.0)	9 (12.1)	4 (15.4)	
Yes, n (%)		87 (87.0)	65 (87.8)	22 (84.6)	
Day job	96				0.89 #
No, n (%)		38 (39.6)	28 (40.0)	10 (38.5)	
Yes, n (%)		58 (60.4)	42 (60.0)	16 (61.5)	
House vs. Apartment	97				**<0.001** #
House, n (%)		66 (68.0)	57 (78.1)	9 (37.5)	
Apartment, n (%)		31 (32.0)	16 (21.9)	15 (62.5)	
Area	97				0.17 #
<100 m^2, n (%)		37 (38.1)	25 (34.3)	12 (50.0)	
>100 m^2, n (%)		60 (61.9)	48 (65.8)	12 (50.0)	
Diagnosis	100				0.39 #
Meningeoma, n (%)		7 (7.0)	6 (8.1)	1 (3.9)	
Astrocytoma, n (%)		35 (35.0)	24 (32.4)	11 (42.3)	
GBM, n (%)		31 (31.0)	26 (35.1)	5 (19.2)	
Oligodendroglioma, n (%)		15 (15.0)	11 (14.9)	4 (15.4)	
Others*, n (%)		12 (12.0)	7 (9.5)	5 (19.2)	
WHO brain tumor grading§	101				**0.023** #
Low grade (WHO I + II), n (%)		50 (50.0)	42 (56.8)	8 (30.8)	
High grade (WHO III + IV), n (%)		50 (50.0)	32 (43.2)	18 (69.2)	
Ongoing therapy	101				0.74 #
No, n (%)		68 (68.0)	51 (68.9)	17 (65.4)	
Yes, n (%)		32 (32.0)	23 (31.1)	9 (34.6)	
**All questionnaires**	**n**	**All (n = 729)**	**Münster (n = 549)**	**Bochum (n = 180)**	***p***
Physical exercise frequency	729				**<0.001** #
Occasionally (<1/week), n (%)		179 (24.6)	109 (19.9)	70 (38.9)	
Often (≥1/week), n (%)		550 (75.5)	440 (80.1)	110 (61.1)	
Social contacts / week	729				0.16 #
0–3, n (%)		227 (31.1)	175 (31.9)	52 (28.9)	
4–6, n (%)		186 (25.5)	137 (25.0)	49 (27.2)	
7–10, n (%)		136 (18.7)	94 (17.1)	42 (23.3)	

10+, n (%)		180 (24.7)	143 (26.1)	37 (20.6)	
HADS–Depression, median (IQR)	729	6 (3–9)	6 (3–9)	5 (4–8)	0.13 [x]
HADS–Anxiety, median (IQR)	729	8 (5–10)	8 (5–10)	7 (5–10)	0.15 [x]
Distress Thermometer, median (IQR)	729	6 (4–8)	6 (4–8)	6 (4–8)	0.96 [x]
WHO5, median (IQR)	729	48 (32–72)	48 (28–72)	48 (34–70)	1.00 [x]

SD standard deviation; IQR interquartile range; BMI body mass index; WHO World Health Organization; HADS Hospital Anxiety and Depression Scale; *Other tumors include neurinoma (n = 3), plexus papilloma (n = 3), ependymoma (n = 2), solitary fibrous tumor (n = 2), germinoma (n = 1), ganglioglioma (n = 1); [§] most recent grading as determined by a neuropathologist; [+] t test; [#] $\chi 2$ test; [x] Mann-Whitney U test.

Table S4. Correlation between outcome parameters among all 729 questionnaires. Spearman's rho and respective p value are given for all correlations.

**Spearman correlations (n = 729)**	**HADS (Depression)**		**HADS (Anxiety)**		**Distress thermometer**	
	**ϱ**	***p***	**ϱ**	***p***	**ϱ**	***p***
HADS (Anxiety)	0.68	<0.001				
Distress thermometer	0.45	<0.001	0.50	<0.001		
WHO 5	–0.71	<0.001	-0.64	<0.001	–0.49	<0.001

Table S5. Correlation between outcome parameters among all 729 questionnaires. Spearman's rho and respective *p* value are given for all correlations.

**Week**	**Number of participants**	**Number of patients**	**Number of relatives**
1	63	40	23
2	55	38	17
3	72	48	24
4	66	43	23
5	59	38	21
6	65	43	22
7	59	39	20
8	57	39	18
9	62	41	21
10	56	39	17
11	58	37	21
12	57	39	18
Median	59	39	21
Interquartile range	57–64	38.5–42	18–22.5

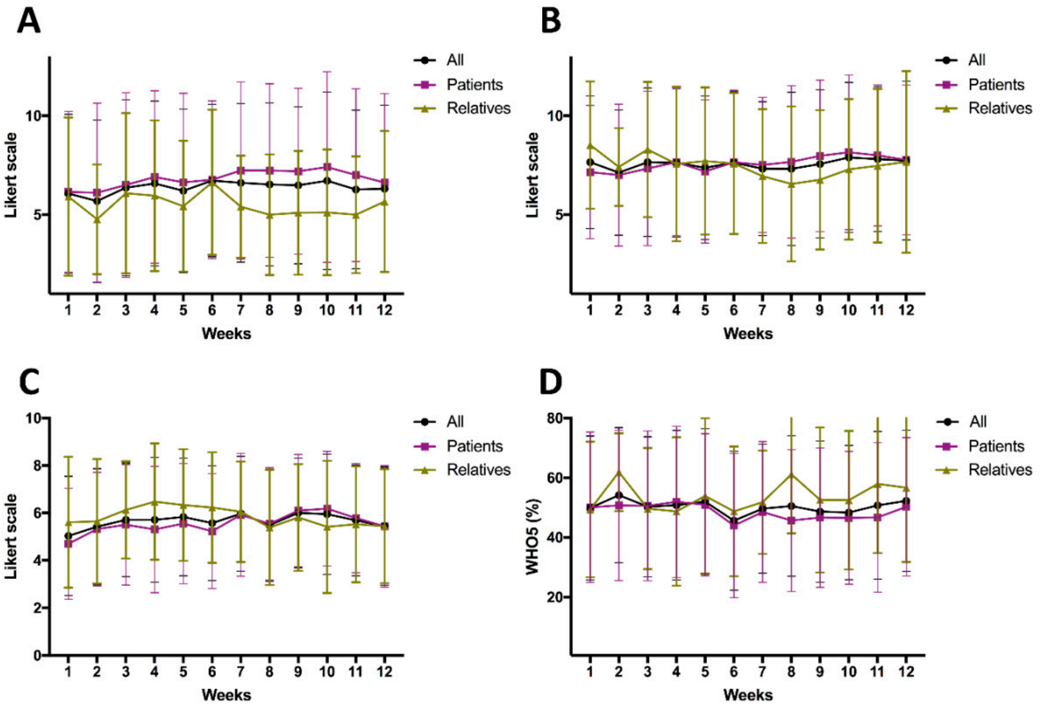

Figure S1. Changes over time for the four main outcomes of depressives symptoms and anxiety measured by the Hospital Anxiety and Depression Score (HADS-D: **A**; HADS-A: **B**), the distress thermometer (**C**) and the WHO5 well-being score (**D**), divided by patients and relatives. Data are presented as mean and standard deviation. Between the two groups, relatives tended to report fewer depressive symptoms (**A**) and increased well-being (**D**).

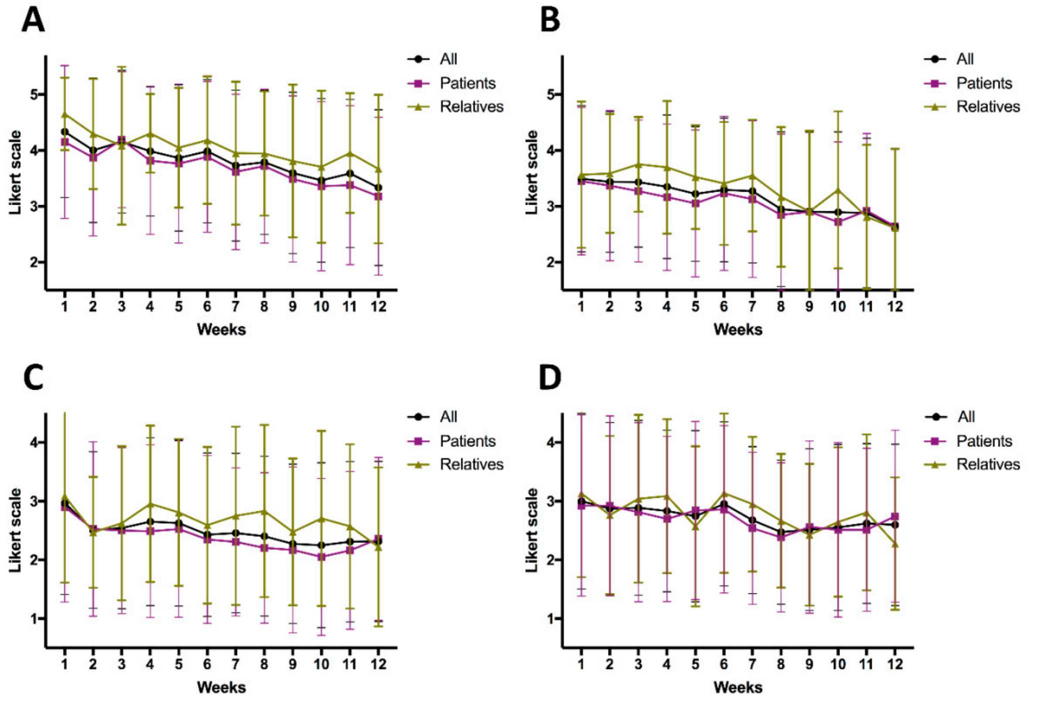

Figure S2. Changes in perception of the isolation over the course of the twelve-week study period. Answers correspond to items 2 (**A**), 3 (**B**), 5 (**C**), and 17 (**D**) from the ISOLA questionnaire, respectively, divided by patients and relatives. Data are presented as mean and standard deviation. Between the groups, relatives tended to report higher understanding for isolation measures, but at the same time were more likely to miss social contacts.

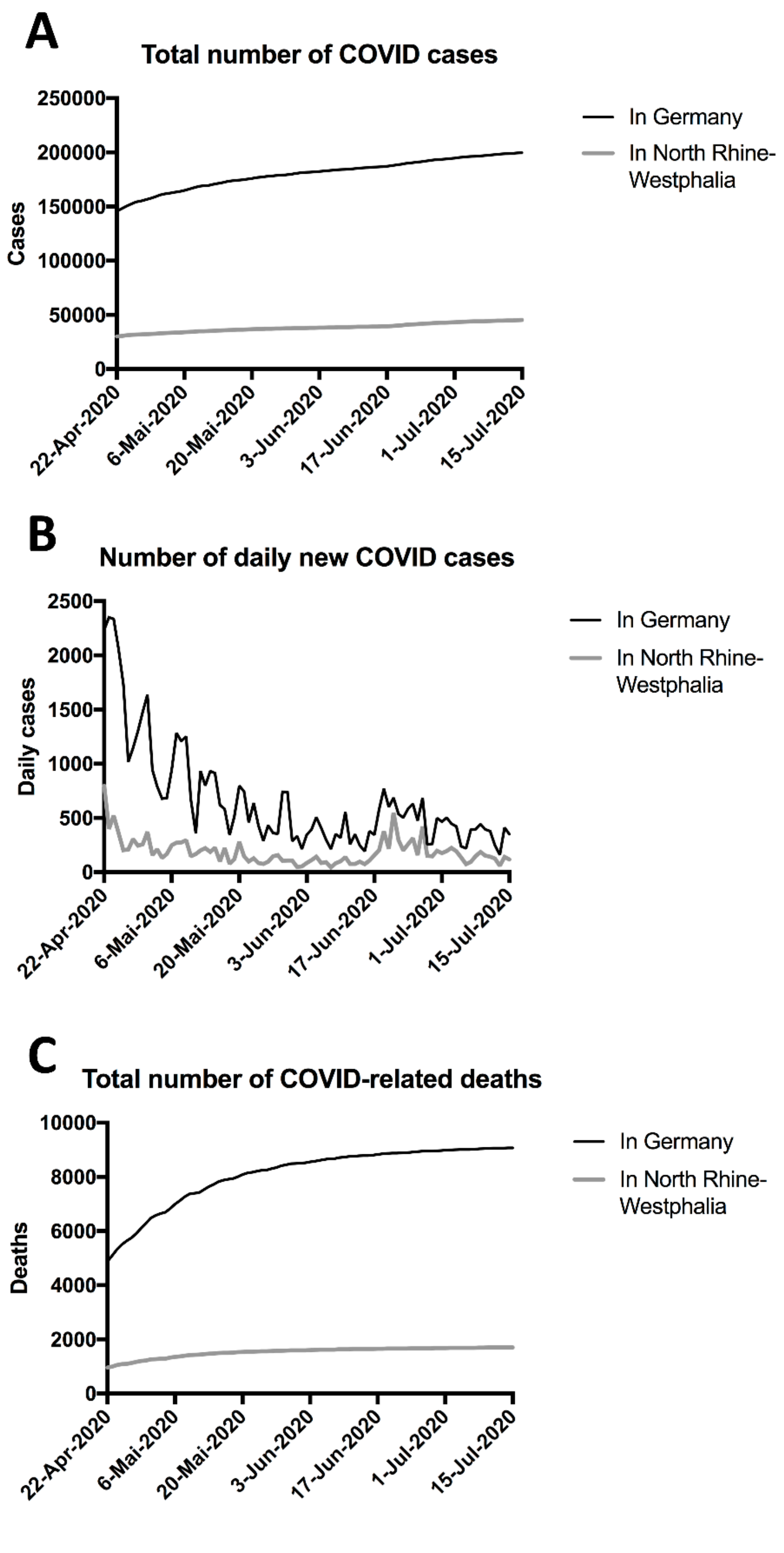

Figure S3. Development of COVID-19 total cases (**A**), daily change (**B**) and COVID-related deaths (**C**). All data was obtained from the Robert-Koch-Institut (source: Robert Koch-Institut (RKI), dl-de/by-2-0) and was graphed by the authors. Use was in accordance with the open data agreement “Open Data Datenlizenz Deutschland – Namensnennung – Version 2.0”.
